# Frailty-aware care: giving value to frailty assessment across different healthcare settings

**DOI:** 10.1186/s12877-021-02722-9

**Published:** 2022-01-03

**Authors:** Kevin F. Boreskie, Jacqueline L. Hay, Patrick E. Boreskie, Rakesh C. Arora, Todd A. Duhamel

**Affiliations:** 1 Faculty of Kinesiology and Recreation Management, University of Manitoba, Winnipeg, Manitoba Canada; 2 Institute of Cardiovascular Sciences, St. Boniface General Hospital Albrechtsen Research Centre, Winnipeg, Manitoba Canada; 3 Max Rady College of Medicine, Rady Faculty of Health Sciences, University of Manitoba, Winnipeg, Manitoba Canada; 4 Department of Emergency Medicine, Max Rady College of Medicine, Rady Faculty of Health Sciences, University of Manitoba, Winnipeg, Manitoba Canada; 5 Department of Surgery, Section of Cardiac Surgery, Max Rady College of Medicine, University of Manitoba, Winnipeg, Manitoba Canada

**Keywords:** Frailty, Emergency Medicine, Critical Care, Primary Care, Perioperative Care

## Abstract

Healthcare systems need to adapt to better serve an aging population with complex presentations. Frailty assessments are a potential means to address this heterogeneity in aging to identify individuals at increased risk for adverse health outcomes. Furthermore, frailty assessments offer an opportunity to optimize patient care in various healthcare settings. While the vast number of frailty assessment tools available can be a source of confusion for clinicians, each tool has features adaptable to the constraints and goals of different healthcare settings. This review discusses and compares barriers, facilitators, and the application of frailty assessments in primary care, the emergency department/intensive care unit and surgical care to cover a breadth of settings with different frailty assessment considerations. The implementation of frailty-aware care across healthcare settings potentiates better healthcare outcomes for older adults.

## Background

People are living longer, however, this longevity often does not equate to years of good health-related quality of life [1, 2]. While aging itself is not a pathological process, biological aging is heterogenous, contributing to diverse patient presentation in healthcare and deviations from what has been traditionally considered the standard patient presentation [1, 3]. Health systems need to adapt to best serve our aging populations [1]. Best care practices may be improved using frailty assessments as tools to better identify complex patients at risk and by personalizing their care using insight beyond chronological age [4, 5].

### Frailty

While no singular definition of frailty has been universally accepted, frailty is generally characterized as a reduced physiologic reserve to adapt to health stressors [6]. Thus, frailty is a predictor of adverse outcomes such as falls [7], hospitalizations [7], morbidity [7–10], and mortality [9–11]. Though frailty prevalence increases with age [12], age and frailty are distinct concepts [3]. Biological age assessed through frailty may be more important than chronological age when assessing risk for adverse health outcomes [9, 13]. A recent meta-analysis of 240 studies from 62 countries by O’Caoimh et al. identified the overall prevalence of frailty ranges from 12 to 24% in community-dwelling adults dependent on the tool used, with prevalence generally increasing with age [14]. This analysis also found a higher prevalence of frailty in females (15–29%) as compared to males (11–20%) [14]. This finding supports prior discussions of the higher frailty prevalence in females despite their greater longevity [15, 16]. Importantly, the identification of frailty status offers a potential opportunity to implement targeted interventions to halt, slow, or reverse health declines, and provides information on the patient that can direct individualized care. The collection of chronological age alone does not offer these opportunities given the heterogeneity seen in aging.

### Frailty assessment

Increasing recognition of frailty assessment as a potential tool in healthcare has led to the development of over 50 frailty tools for a variety of settings [4, 5]. While there is low agreement between many frailty assessments, different tools likely identify different constructs of frailty [17, 18]. Frailty assessments are broadly classified by methodological approach: phenotypic, multidimensional, or an accumulation of deficits. Phenotypic approaches focus on physical clinical criteria including unintentional weight loss, low physical activity levels, and reduced muscle strength as a means of identifying frailty [4, 7]. Critics argue phenotypic approaches may be limited in scope and recommend the incorporation of a wider range of criteria, such as cognitive or psychological factors [4, 19]. To this end, multidimensional approaches have been developed to identify frailty beyond phenotypic presentation [4]. Furthermore, the accumulation of deficits approach seeks to identify frailty by examining a range of deficits (i.e. signs, symptoms, disabilities or disease presence) that would increase risk for adverse outcomes [20].

Frailty assessment is a recommended addition to the clinician toolkit to help identify individuals at risk for health decline aggravated by health stressors [4, 5]. The importance of these frailty assessments in an aging population is evident by the calls to action supporting their incorporation into clinical practice to better predict adverse health outcomes [13, 21, 22]. In fact, the Canadian Frailty Network top ten research priorities focus on frailty assessment as a means for healthcare practitioners to inform treatment and care decisions, as well as to avoid unnecessary hospitalization and emergency department visits for older adults [23]. Implementing frailty assessments in clinical care has the potential to improve patient care [13, 21, 22]. Despite the existence of obstacles to the incorporation of frailty into clinical practice such as uncertainty in tool selection [22, 24], support for the incorporation of frailty across healthcare settings continues to evolve.

**Fig. 1:**
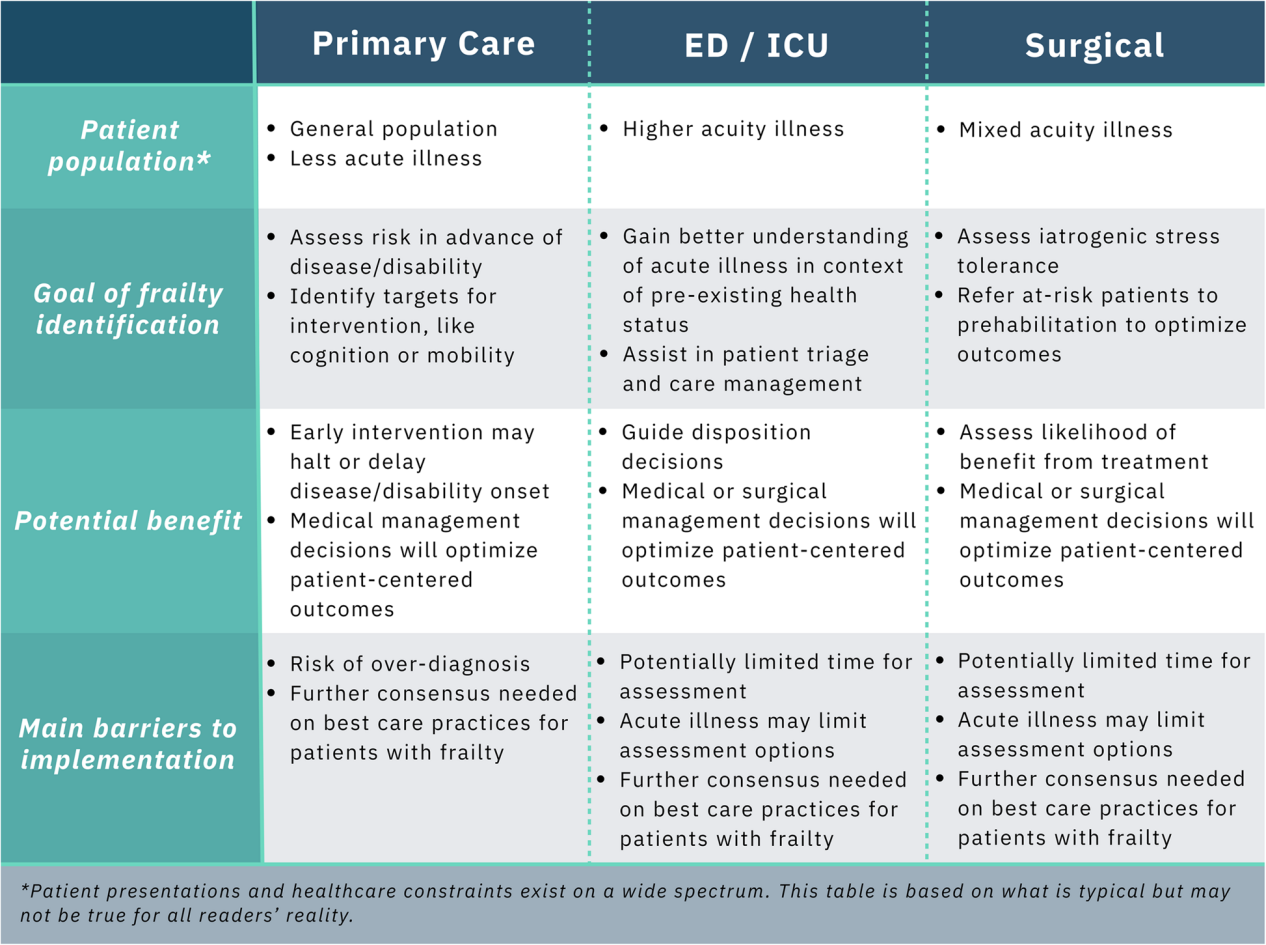
Summary of main considerations for frailty assessment in primary care, acute care and the surgical context

This review is intended to offer a current understanding of the value of frailty assessment in different settings to facilitate the implementation of frailty into practice by clinicians. MeSH terms such as “Frail Elderly”, “Geriatrics”, “Geriatric Assessment/methods” and “Frailty/diagnosis” were first used to identify relevant manuscripts using additional terms specific to the various clinical settings. Thereafter, a forward-and backward search strategy was implemented to identify additional relevant publications. We also used resources such as Connected Papers (www.connectedpapers.com) to identify related works. However, the search strategy was not formally developed or reviewed by a librarian and should not be considered a systematic methodology. Current opportunities and challenges for frailty-aware care will be compared between primary care, the emergency department (ED)/intensive care unit (ICU), as well as the perioperative context [25–27]. Figure 1 summarizes the spectrum of considerations in each setting – human resource constraints, patient population, and outcomes of interest. While these settings do not cover the gamut of clinical practice, the competing interests for frailty assessment in each of these settings are widely applicable given the breadth of practice covered. As opposed to comparing frailty assessment methodologies within each context, this review paper seeks to compare overarching principles in frailty assessment between clinical contexts. Similarly, the frailty assessment tools discussed are not exhaustive, but we intend to highlight some of the most researched approaches related to each clinical setting.

## Main text

### Frailty in primary care

As chronic diseases continue to be a higher attributable cause of death [28] and as the population ages [29], frailty assessment approaches gain merit as a means of directing primary prevention efforts [30]. Primary care serves a critical aspect of prevention by providing longitudinal care for patients. Since physiologic deficits accumulate over time, primary care offers an opportunity to intervene further upstream, potentially before the appearance of clinical symptoms [2, 31]. Healthcare systems are largely based on addressing single-system disease [32] and patient management challenges increase with higher prevalence of multisystem disease [29]. Frailty assessment offers an opportunity to identify those at risk of multisystem downward health trajectories with more complex presentations and to direct therapeutic interventions at components of frailty earlier [33, 34].

#### Applying frailty assessment in primary care

Early intervention is vital, as health trajectories typically accelerate downward with frailty progression and become increasingly difficult to reverse or slow [21]. Thus, an effective frailty tool in the primary care setting should be one that can identify not only those who are already frail, as there is a high prevalence in the community [14], but also those in the early stages of frailty. Patient health trajectories are more amenable to change earlier and a greater number of therapeutic options may be available [35, 36].

Frailty assessments in the primary care setting should also identify targets for intervention, such as health risk behaviours or domains of frailty, like cognition or mobility [37]. Identifying targeted areas for health improvement allows for a personalized care approach and is more apt to pre-emptively improve the status of those who are at risk of frailty progression [35, 37]. For example, individuals with frailty and polypharmacy may benefit from a thorough prescription review [38, 39], while those reporting challenges in activities of daily living may require a physical activity intervention to improve functional status [40, 41]. Currently, identifying optimal interventional strategies are challenging given varied frailty definitions and assessments, though multi-modal interventions involving physical activity and nutrition have demonstrated promise [35].

Validated frailty assessments for primary care vary in the ease of administration, with more intensive options likely requiring allied healthcare staff beyond the physician to save cost and time. In comparison, rapid options may require the referral of patients in need of intensive screening through more thorough methodologies with a multidisciplinary team [42–44]. The International Conference on Frailty and Sarcopenia Research Consensus Guidelines recommend referral to a CGA after initial assessments identifying frailty given the low specificity often observed in frailty screening [44, 45]. A careful balance must be struck between the efficacy of the frailty approach used to identify interventional targets and the feasibility of utilizing the approach within resource constraints.

Importantly, frailty assessment in the primary care setting also potentiates improved clinical decision making for complex patients [30]. For example, the pharmaceutical management of hypertension in adults with frailty is under reconsideration [46, 47]. Newer recommendations from experts suggest higher target blood pressure values for hypertensive adults with frailty than those who are robust, as aggressive treatment in those who are frail may lead to adverse outcomes [46, 47]. The direction of care and shared clinical decision making with regards to frailty status requires communication between patient and physician. Communicating effectively on the topic of frailty with patients in the primary care setting requires a shared understanding of the concept with a focus on person-centered communication [48].

The incorporation of frailty assessment will require justifiable time, personnel, and technology to incorporate into primary care. In spite of the initial increased resource costs of frailty assessments, research indicates long-term cost-effectiveness and improved patient outcomes are possible and justified [35, 49]. As opposed to considering frailty a case of over-medicalization, leading geriatricians argue frailty assessments should be a part of general care and have likened the careful monitoring of frailty status to that of monitoring hypertension to reduce risk of myocardial infarction [21]. Similarly, frailty status should be identified, treated, and monitored over time on a continuum as a means of reducing risk for disability and disease [21].

#### Examples of frailty assessment in primary care

Frailty tools utilized in the primary care setting should have the ability to identify intervenable aspects of health and can identify those in need of increased healthcare attention. The following tools are far from an exhaustive list of recommended tools for this setting but can give insight on the variety of approaches available as well as some of their advantages and disadvantages.

The Fried physical frailty phenotype [7] (PFP) is one of the most commonly cited frailty assessments in the literature which focuses on five phenotypic aspects of frailty: 1) muscle weakness; 2) slow walking speed; 3) low physical activity levels; 4) unintentional weight loss; and 5) self-reported exhaustion. Frailty status is then assigned based on a total score ranging from zero to five (i.e. 0 = robust; 1–2 = pre-frail; ≥ 3 = frail) dependent on the number of criteria present as assessed through a combination of brief questionnaires and physical function testing, such as grip strength and gait speed. Phenotypic approaches, such as the PFP, are useful for identifying physical deficits and/or potential nutritional deficits in a patient but are potentially limited by only examining physical presentation [4, 19]. General signs of phenotypic decline do not provide direction for preventative or therapeutic strategies without then identifying root causes, but their collection has the benefit of not requiring preliminary clinical evaluation [50]. Frailty assessments with physical components also have the limitation of requiring patients fit enough to complete the testing [51], although inability to complete functional testing can also sometimes be informative. Additionally, physical assessments often take more time, lowering the feasibility of implementation [17, 50].

Examining frailty as an accumulation of deficits, the Frailty Index [52] (FI) approach has been suggested as a means of frailty assessment applicable to a variety of clinical settings including primary care [22, 32, 53, 54]. An FI ratio between zero and one is determined by calculating the number of existing deficits presented, divided by the total number of deficits assessed in the patient [52]. Typically, deficits include clinical signs, symptoms, disabilities, morbidities, and/or laboratory tests that relate to health status and represent a broad range of physiological systems [52] – an approach that has the potential to identify specific intervenable domains of health [20]. The approach has been criticized for being cumbersome given an FI typically includes 35 or more deficits [52], lowering its feasibility of implementation [17]. Creative uses of the FI approach have been introduced in the form of an electronic FI (eFI) that uses data already stored in electronic health records to create a score that could be automatically calculated for use in primary care [54, 55] as well as FIs that use less variables and focus on common clinical and laboratory tests (FI-Lab) [56].

The Fatigue, Resistance, Ambulation, Illnesses & Loss of Weight (FRAIL) scale [57] is also a phenotypic frailty assessment but it is administered entirely via a brief questionnaire that examines self-reported fatigue, resistance, ambulation, illness, and loss of weight [57]. The FRAIL scale is easy to use [17] and has been validated in a variety of populations and has predictive ability for adverse health outcomes [42, 57, 58]. Its use has been recommended as a tool to stratify those in need of a Comprehensive Geriatric Assessment (CGA) to identify specific targets for intervention [42].

The Kihon Checklist [59] is a multidimensional frailty assessment widely used in Japan that consists of 25 yes/no questions covering categories like nutrition, activities of daily living, activity, socialization and cognition. The results of this rapid checklist correlate closely with other validated tools assessing frailty phenotypes [60] and it has been shown to predict dependency and mortality [61]. Similarly, the validated Tilburg Frailty Indicator (TFI) uses a rapid self-report questionnaire to assess physical, psychological and social domains of frailty [62] to predict adverse outcomes like healthcare utilization and falls [63].

In contrast to the FRAIL scale and the Kihon Checklist based solely on questionnaire, the multidimensional Edmonton Frail Scale (EFS) uses a combination of questionnaire and assessment to assess functional, health and social factors while incorporating cognitive assessment through clock drawing and functional performance with a timed up and go test [64]. This tool has also been associated with adverse outcomes such as mortality [65].

### Frailty in the emergency department/intensive care unit

Patient illness in the ED and ICU is typically acute in nature, making time per patient and patient flow necessary considerations. As such, patient triage and rapid decision-making tools validated in these environments are of importance. Using the best available clinical evidence, physicians must be able to assess the risk-benefit of various treatments, as well as have informed discussions regarding patient goals of care. Assessment and decision tools to assist staff in the ED/ICU setting to triage, care, and allocate resources are needed [66], especially for an aging population presenting with increasing case complexity and comorbidities [67]. Currently, triage models mistakenly under-triage older adults and provide interventions that may not yield benefit for the patient [68, 69] or simply cannot identify vulnerable older adults [70]. Frailty assessment has been suggested to allow for a better understanding of patient presentation relative to their baseline health status [34].

#### Applying frailty assessment in the ED/ICU

Frailty assessment has been proposed as a potential means of improving triage methods and identifying appropriate care for older patients in the ED/ICU [26, 71–74]. Given that patients with frailty are more likely to experience adverse outcomes, either through presenting illness or iatrogenic stress, care has to be adapted to meet the patient needs [75]. Recent systematic reviews have identified frailty as a predictor of in-hospital mortality, length of hospital stay, subsequent nursing home admission, and mortality in hospitalized older adults [76, 77]. Frailty assessment could assist with facilitating referrals to various health services and identifying optimal treatment options for that patient [69]. In severe illness, the additional iatrogenic stress of intensive care interventions needs to be considered in patients with advanced frailty [78]. Furthermore, frailty status affects the association between acuity of patient illness and mortality [79, 80]. For example, Pulok et al. found that high acuity illness was associated with significant mortality risk regardless of frailty status, but even those with low acuity illness were at risk for mortality when the degree of frailty was higher [79].

The context of emergency and critical care necessitates considering different frailty tools than those best for primary care - validity must be carefully balanced with the feasibility of implementing an assessment in an acute care environment [77, 81]. A number of tools have been specifically developed and have demonstrated feasibility in urgent care, where time restraints on the clinical team and patient presentation limit the ability to perform certain tests [82]. In the ED, many patients may be unable to perform common phenotypic frailty assessments, such as grip strength or gait speed, rendering brief assessments or clinical judgement more appealing [44]. Unfortunately, some rapid tools specifically designed for the ED, such as the Triage Risk Screening Tool [83] (TRST) and Identification of Seniors At Risk [84] (ISAR) tool have demonstrated poor prediction of adverse outcomes following ED encounters with older adults for outcomes like ED returns and functional decline [70].

The selection of useful tools is dependent on choosing appropriate goals for frailty identification. In the emergency and critical care settings, frailty assessments namely identify optimal treatment and/or triage as well as inform shared decision making. Furthermore, frailty assessment in critical care may also lead to subsequent referrals to additional health services to better support the patient long-term [69, 81]. Theou *et al.* have recommended that frailty assessments in the ED/ICU could prevent premature case closure, facilitate the creation of multi-disciplinary care plans prior to discharge, and give an indication of cases involving more than a single pathology [69]. Early assessment of frailty status at the ED can allow for the timelier initiation of a thorough CGA that can be used to inform patient discharge [74], such as the implementation of homecare supports. Rapid frailty assessments are potentially efficacious for this purpose, as long as they are able to predict adverse patient outcomes, can assist in optimizing treatment, and result in better informed decisions regarding goals of care.

#### Examples of frailty assessment in the ED/ICU

Though more thorough assessments could potentially be conducted upon admission to a critical care ward in the hospital, initially, rapid frailty assessments are recommended in the context of the ED/ICU [81, 82, 85]. Each rapid frailty assessments tool has inherent limitations but offers the flexibility and ease of measurement required for use in the ED/ICU.

The Clinical Frailty Scale (CFS) has been suggested as one of the more studied approaches to assess frailty in the ED/ICU [53, 85, 86] and has demonstrated association with mortality, length of stay, ICU admission, and readmission [87–89]. It is the only tool to date that has been found to be valid and reliable for these outcomes in the acute care setting [89]. The CFS has convergent validity with the FI and Fried approaches as well as measures from the CGA [71, 87, 90]. Frailty status is assessed on geriatric clinical judgement of mobility impairments, function, and cognition from two weeks prior making it a multidimensional approach. This information can also come from collateral sources, such as family members [86], which would be particularly important in the intubated or obtunded patient. The CFS is only recommended for use in adults over the age of 65 years [86].

The Hospital Frailty Risk Score (HFRS) was developed in an attempt to avoid any inter-rater or response bias errors of a rapid assessment tool [66]. International Statistical Classification of Diseases and Related Health Problems, Tenth Revision (ICD-10) codes are used to calculate the HFRS. ICD-10 codes identify the illnesses and injuries that have been claimed on hospital billing [91], making it feasible to implement the HFRS into existing hospital information systems. Moreover, the HFRS has been validated in multiple cohorts and has moderate agreement with the Fried and FI approaches [66].

The FI model of frailty assessment [87] has also been recommended in the acute care context, albeit the approach is modified slightly to better suit the ED/ICU [26]. The same conceptual creation of a FI using a ratio of existing to total deficits can still be used in this setting [26, 52]. For example, the FI-ED utilizes variables easily measured and/or accessible in the ED [26]. Despite having only 24 variables, the FI-ED was able to identify increased risk for admission, prolonged hospital stay, discharge to long-term care, and mortality at 28 days [26]. Although FI-Labs have been developed [56, 92] and used in the hospital setting [93], the design of these approaches would have to take precaution not to use measures that may be saturated in the typical patient presenting to critical care [52]. FI-Labs used in acutely hospitalized older patients have been associated with mortality post discharge [94, 95].

Future research to better support the increasing number of complex patients with frailty presenting to the ED/ICU is needed. Further research is required to facilitate a greater understanding of the feasibility, convergent validity, and predictive validity of varied frailty assessments [82, 96]. Considerations for frailty assessment in the ED/ICU setting are summarized in Fig. 1.

### Frailty in the surgical context

Advances in medicine have led to the surgical referral of older patients [97]. However, some patients are less equipped to handle the iatrogenic stress associated with invasive surgery [97–99]. Enhancing recovery protocols for various surgical procedures have been developed in recognition that changes to clinical practice are needed to improve surgical outcomes [100]. These approaches have re-examined perioperative management to improve patient outcomes.

As in the ED/ICU setting, identifying patients at risk for adverse outcomes could be valuable in assessing the likelihood of benefit of surgical intervention and potentiates referrals to interventions aimed at increasing patient resilience prior to surgery [101–103]. If a patient with frailty is known to be less able to tolerate health stressors such as an invasive surgery, less invasive options or even non-surgical supportive therapy may better fit the goals of patient care. For example, surgical aortic valve replacement (SAVR) is the choice of intervention for many patients requiring valve replacement. The less invasive transcatheter aortic valve implantation (TAVI) is used in those at higher risk of surgical and post-surgical adverse events [104]. An assessment of frailty status could be used as a means of making the most informed choice for surgical intervention [104, 105] and could also guide perioperative care, such as tailoring the anaesthetic or other specific approaches to prevent delirium onset [73] of which frail individuals are at increased risk [106].

Cardiac surgery research and care has strongly adopted frailty assessment as a means to improve patient care [107]. Increasingly, older patients at risk for frailty are being referred for consideration of procedures like coronary artery bypass grafts and TAVI [108]. Frailty status increases patient risk for adverse cardiac surgical outcomes [25, 109–112], and this led to the recommendation of prehabilitation to improve functional status in patients awaiting surgery [113]. The potential for prehabilitation approaches to improve patient outcomes in cardiac procedures is promising [114–116], but further randomized controlled trials are needed to assess feasibility, cost-effectiveness and efficacy in this and other surgical contexts [103]. Importantly, preoperative frailty status has also identified individuals who are less likely to attend cardiac rehabilitation after cardiac surgery [117] – a rehabilitation approach that is known to reduce adverse events postoperatively [118] and may further improve frailty status [119].

#### Applying preoperative frailty assessment

Frailty is recommended as an assessment of patient resiliency in the preoperative period [73, 98]. Increasing complexity of surgical patients with comorbidities and functional limitations has meant that frailty assessment increasingly provides additional utility to current methods of risk stratification preoperatively [120]. In a recent systematic review, frailty status was associated with surgical complications, readmission, and mortality across a wide variety of surgical procedures [121]. While severity of illness of the surgical patient is quite dependent on the procedure, physical limitations and acuity of presentation may restrict possible frailty testing. Elective surgeries, for the most part, would be less urgent than the interventions for a patient in the ED/ICU, leaving more time for thorough frailty assessment. Concern has previously been raised, however, regarding the implementation of performance-based testing in the outpatient setting given the additional workload and the feasibility of performing such tests in all populations [65].

#### Examples of preoperative frailty assessment

Due to flexibility and thorough validation, the FI approach has been suggested for use in the preoperative setting [121, 122]. Simplified FIs with less variables and easily collected data have been recommended [98, 101]. For example, a modified FI (mFI) has been created using a simplified FI designed with only 11 variables and has been tested in surgical settings [123, 124]. A recent systematic review of preoperative frailty assessment identified FI assessments as being feasible while demonstrating strong predictive validity for surgical complications [122].

The Modified PFP and the CFS have been identified as tools that could be used to identify those at risk of reduced quality of life and mortality [122, 125]. Interestingly, further analysis in one study used these scores to improve the ability of existing surgical risk tools, specifically the European System for Cardiac Operative Risk Evaluation II which discriminates patients at risk of poor functional survival [125]. As in other clinical contexts, there is some concern about the ability of frail preoperative patients to perform some phenotypic frailty assessments [73, 122]. It has been recommended that protocols should utilize scoring systems for frailty assessment that do not exclude patients who are unable to perform the assessment [25].

The Essential Frailty Toolset (EFT) has been specifically recommended by Afilalo et al. as the optimal tool for the preoperative cardiovascular surgery assessment of patients being considered for TAVI or SAVR [126]. The EFT can be rapidly assessed, using only a 4-item scale that includes: 1) lower extremity weakness; 2) cognitive impairment; 3) anemia, and; 4) hypoalbumineria. In the FRAILTY-AVR Study, the EFT was the strongest predictor of worsening disability and mortality at both 30 days and one year as compared to the PFP, FI, and Short Performance Physical Battery, to name a few [126]. However, the EFT has yet to be tested in other surgical contexts.

Despite the promising research advocating for frailty assessment in perioperative practice, frailty assessment has yet to become widely adopted. Frailty assessment should be strongly considered for standard preoperative care to identify those who are less likely to receive benefit from invasive procedures and to identify those who should receive prehabilitation prior to surgical intervention [98, 127]. Considerations for frailty assessment in the preoperative setting are summarized in Fig. 1.

### Moving toward clinical implementation of frailty

Successful adoption of frailty into clinical practice requires further consensus on both the definition and optimal assessment methodology for the proposed contexts [22]. Optimal frailty assessment for various clinical settings, in turn, is dependent on the inherent constraints and the goals of use in each setting [17]. While there is no consensus of assessment for each individual setting, work such as that by Oviedo-Briones et al. [17] and Aguayo et al. [18] comparing the feasibility and application of various tools in a variety of settings is necessary to achieve this. These works have both found low agreement between many frailty assessments and conclude that different tools are likely assessing different aspects of frailty or that variable subtypes of frailty exist [17, 18]. Thus, tools should be selected based on feasibility and on their ability to identify the components of frailty important for that specific setting. Further work is needed to identify optimal approaches for acting on this information in those identified as being frail either through personalized care management, the identification of goals of care or interventional approaches. Clinical settings identify not only the need for intervention addressing frailty status, but also for frailty-informed care management [69, 73, 81, 98].

Further consensus on tool selection in various healthcare settings will allow for the refinement of individualized care plans to best care for those identified as patients at increased risk for adverse outcomes based on their frailty status and to improve their resilience [21, 22]. The implementation of frailty assessment and intervention in the clinical setting has, in part, been hampered by the incorrect use of presumed synonyms for frailty such as sarcopenia and disability, as well as the continual influx of new methodological approaches and tools [22] that may be assessing different components of frailty [17]. Arguably, the most effective interventions are multi-dimensional, as the most successful interventions to date have included aspects of both physical activity and dietary intervention [35, 128], as frailty itself is indicative of reduced physiologic reserve across a range of systems. Identifying an individual living with frailty could lead to subsequent CGA to provide more comprehensive information and to direct ongoing intervention [44]. Current interventions typically focus on components of frailty [35], but a further understanding of the broad pathophysiology seen in frailty as well as clinically meaningful changes [129] in frailty status are needed in order to address this syndrome of physiologic decline.

**Fig. 2:**
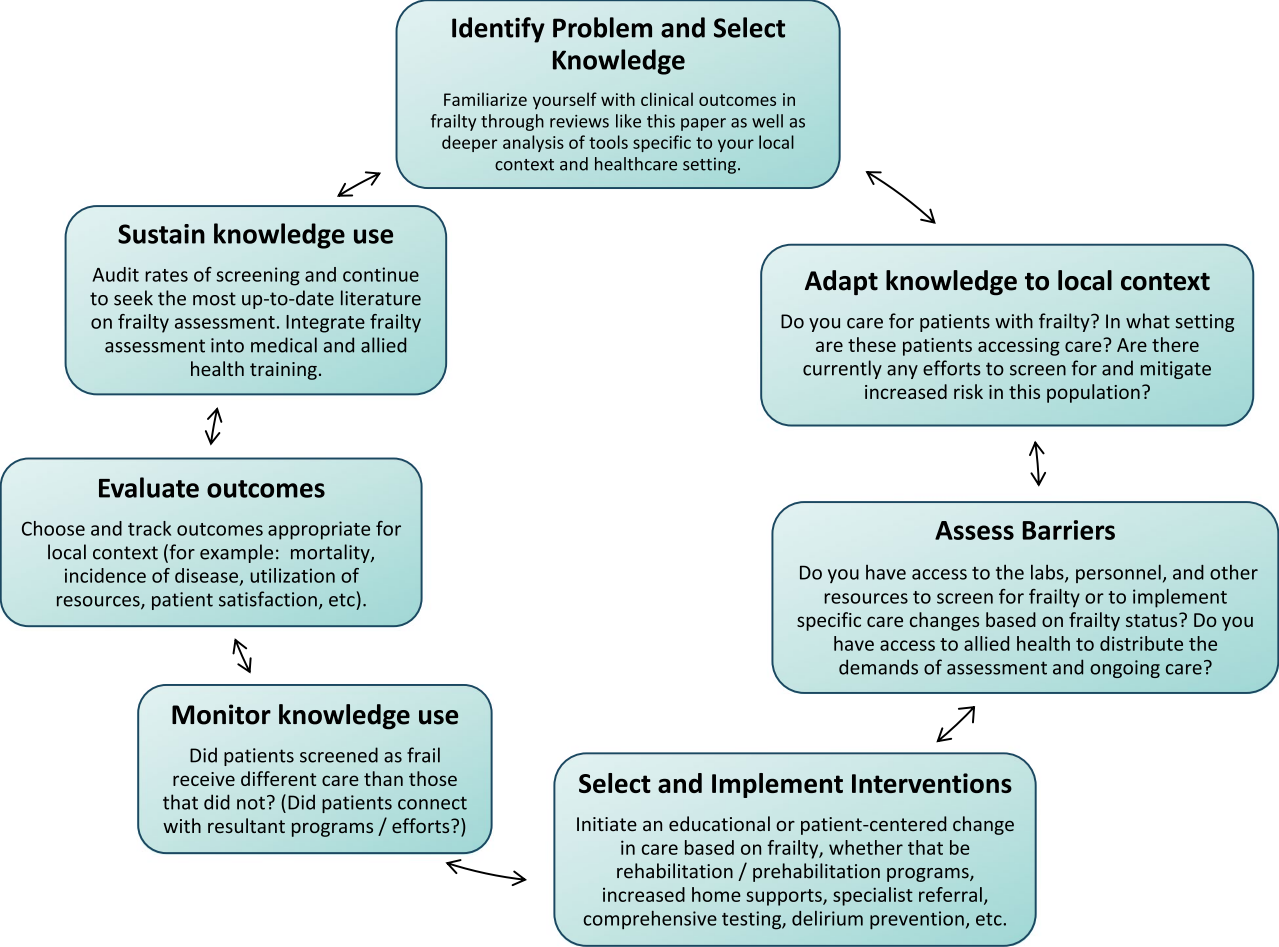
Knowledge-to-action cycle for incorporating frailty into clinical settings. *Based on the KTA cycle outlined by Graham et al.* [130]

Initial analyses in the primary care setting have suggested that frailty-focused intervention, as opposed to usual care, provides better care and better patient outcomes without increasing costs [35]. Cost-analyses will have to be continually explored as new interventions and individualized care pathways are identified in expanding other clinical contexts, as in the ED/ICU [74, 81] and in preoperative care [121]. Further research is needed in order to identify the most efficacious approaches for therapeutic interventions in populations with frailty based on outcomes supported by the patient themselves, as well as to guide best care practices that take advantage of frailty identification in the clinical setting to support patient health [73]. Supporting patient health through the identification of frailty status requires a further understanding of effective patient-physician communication on the topic [48]. Future research on the use of frailty assessment in clinical settings should address this challenge and should use patient-oriented research designs to insure that the questions addressed are important to those living with frailty, such as the Top Ten Frailty Priorities developed by the Canadian Frailty Network [23]. Advice regarding the specific implementation of frailty-aware care will be unique to local context and healthcare setting, so we advise using a Knowledge-to-Action (KTA) framework to determine steps forward (Fig. 2). The KTA framework outlined by Graham *et al.* presents seven bi-directional steps to appropriately integrate knowledge while examining knowledge gaps and problems [130].

## Conclusions

Barriers to implementing frailty assessments in clinical settings still exist - namely a lack of consensus on the assessment tool best suited in each context, uncertain cost-effectiveness, and few established interventions for frailty [21, 22, 30, 32]. What is known, however, is the significant health burden that frailty presents [13, 49]. Frailty costs our healthcare system [34, 49] in terms of increased hospital admissions, specialized consultations, longer hospital stays, and adverse outcomes [34, 131]. Adding frailty assessments into clinical practice could allow for further refinement of its effectiveness for directing individualized care pathways and practicality for implementation into a variety of clinical settings [21, 30]. Current healthcare systems are not designed to manage the complexity of patient presentation with multimorbidity that may become more common with an aging population [33]. Though the integration of frailty assessment into clinical care is not yet widely accepted, the emerging body of literature is sufficient to guide assessment implementation for a variety of medical settings as a step towards frailty-aware care that could improve acute and longitudinal patient treatment by guiding individualized care decisions and identifying the need for potential additional resources throughout care. Medicine has to shift with the aging demographic and changing presentation of the patient populations it is meant to help [1]. The implementation of frailty assessment across a variety of healthcare settings may get us closer to achieving better healthcare outcomes for older adults.

## Data Availability

Not applicable.

## Abbreviations

CFS: Clinical Frailty Scale
CGA: Comprehensive Geriatric Assessment
ED: Emergency Department
eFI: Electronic Frailty Index
EFS: Edmonton Frail Scale
EFT: Essential Frailty Toolset
FI: Frailty Index
FRAIL: Fatigue, Resistance, Ambulation, Illnesses & Loss of Weight
HFRS: Hospital Frailty Risk Score
ICD-10: International Statistical Classification of Diseases and Related Health Problems, Tenth Revision
ICU: Intensive Care Unit
ISAR: Identification of Seniors At Risk
mFI: Modified Frailty Index
PFP: Physical Frailty Phenotype
SAVR: Surgical Aortic Valve Replacement
TAVI: Transcatheter Aortic Valve Implantation
TFI: Tilburg Frailty Indicator
TRST: Triage Risk Screening Tool
